# Changes in the Presentation of Infectious Disease Data in the National Notifiable Diseases Surveillance System — January 2015

**Published:** 2015-01-30

This issue of *MMWR* incorporates changes to Table I (Provisional cases of selected infrequently reported notifiable diseases [<1,000 cases reported during the preceding year], United States) and Table II (Provisional cases of selected notifiable diseases [>1,000 cases reported during the preceding year] and selected low frequency diseases, United States). This year, the Table I and Table II modifications add conditions designated or proposed as nationally notifiable by the Council of State and Territorial Epidemiologists (CSTE) and CDC (1–5). In addition, the presentation of viral hemorrhagic fevers data in Table I reflects recent enhancements made to the National Notifiable Diseases Surveillance System (NNDSS) to enable reporting jurisdictions to submit electronic case notifications for specific viral hemorrhagic fevers.

## Modifications to Tables I and II

Campylobacteriosis has been added to the list of nationally notifiable infectious diseases and conditions. Incidence data for campylobacteriosis will appear in Table II. CSTE requested chikungunya virus disease, dengue-like illness, and non-hantavirus pulmonary syndrome (non-HPS) hantavirus infection be added to the list of nationally notifiable infectious diseases and conditions. (In the past, HPS has been nationally notifiable, but hantavirus infections not complicated by the pulmonary syndrome were not.) Incidence data for chikungunya virus disease and “hantavirus infection, non-HPS” will appear in Table I, whereas dengue-like illness will appear in Table II, after CDC obtains Office of Management and Budget Paperwork Reduction Act approval to receive data for these conditions. The national surveillance case definitions for these diseases and conditions are listed in their respective CSTE position statements (2–4) and are posted in the case definitions section of the NNDSS website (1). Three low-incidence conditions (rubella, rubella congenital syndrome, and tetanus) have been moved from Table I to Table II to facilitate monitoring incident case counts by reporting jurisdiction. Vibriosis also has been moved to Table II; the number of cases reported for this condition during each of the previous 3 years was >1,000.

Previously, NNDSS did not receive electronic data about incident cases of specific viral hemorrhagic fevers; instead, data were collected in aggregate and reported in Table 1 as “Viral hemorrhagic fevers.” In response to the need to monitor viral hemorrhagic fevers separately, beginning January 1, 2015, cases of Crimean-Congo hemorrhagic fever, Ebola hemorrhagic fever, Guanarito hemorrhagic fever, Junin hemorrhagic fever, Lassa virus infection, Lujo virus infection, Machupo hemorrhagic fever, Marburg fever, and Sabia-associated hemorrhagic fever will be reported separately.
